# Bovine lactoferrin free of lipopolysaccharide can induce a proinflammatory response of macrophages

**DOI:** 10.1186/s12917-016-0878-2

**Published:** 2016-11-10

**Authors:** Nada Zemankova, Katarina Chlebova, Jan Matiasovic, Jana Prodelalova, Jan Gebauer, Martin Faldyna

**Affiliations:** 1 Veterinary Research Institute, Hudcova 296/70, Brno, 621 00 Czech Republic; 2 Faculty of Science, Masaryk University, Brno, Czech Republic

**Keywords:** Inflammatory cytokines, LPS, NFκB, NIK, TLR4

## Abstract

**Background:**

Lactoferrin (LF) is an 80 kDa glycoprotein which is known for its effects against bacteria, viruses and other pathogens. It also has a high potential in nutrition therapy and welfare of people and a variety of animals, including piglets. The ability to bind lipopolysaccharide (LPS) is one of the described anti-inflammatory mechanisms of LF. Previous studies suggested that cells can be stimulated even by LPS-free LF. Therefore, the aim of our study was to bring additional information about this possibility. Porcine monocyte derived macrophages (MDMF) and human embryonic kidney (HEK) cells were stimulated with unpurified LF in complex with LPS and with purified LF without bound LPS.

**Results:**

Both cell types were stimulated with unpurified as well as purified LF. On the other hand, neither HEK0 cells not expressing any TLR nor HEK4a cells transfected with TLR4 produced any pro-inflammatory cytokine transcripts after stimulation with purified LF. This suggests that purified LF without LPS stimulates cells via another receptor than TLR4. An alternative, TLR4-independent, pathway was further confirmed by analyses of the NF-kappa-B-inducing kinase (NIK) activation. Western blot analyses showed NIK which activates different NFκB subunits compared to LF-LPS signaling via TLR4. Though, this confirmed an alternative pathway which is used by the purified LF free of LPS. This stimulation of MDMF led to low, but significant amounts of pro-inflammatory cytokines, which can be considered as a positive stimulation of the immune system.

**Conclusion:**

Our results suggest that LF’s ability is not only to bind LPS, but LF itself may be a stimulant of pro-inflammatory pathways.

## Background

Lactoferrin (LF) is an 80 kDa iron-binding glycoprotein that plays an important role in the innate immune system [1–7]. It is also a multifunctional protein with a supportive therapeutic potential against bacterial, fungal or viral infections [8–10]. At present, LF is the focus of a variety of research areas due to its anti-inflammatory and anti-cancer properties, and the resulting therapeutical potential [11–14]. It was found that LF interacts with LPS and then activates NFκB via TLR4 pathway [15]. This suggests that immunomodulatory effects of LF could be due, in part, to LPS binding [16, 17]. To date, it is known that bovine LF affects the innate immunity by activating specific events in macrophages through interaction with TLR4-dependent and TLR4-independent pathways. It was found that bovine LF function is not dependent on TLR4 with respect to IL-6 production [18]. Since LF has a broad spectrum of actions, it also uses a wide range of signaling pathways for this purpose. In addition to NFκB, LF affects also the MAP kinase pathway. There, activation of ERK1/2 as well as of p38 stress-activated kinase has been reported [19, 20].

NF-kappa-B-inducing kinase also known as Mitogen-activated protein kinase 14 (NIK) is encoded by the MAP3K14 gene which is a serine/threonine protein kinase [21]. NIK belongs to the group of relevant activators of the NFκB alternative pathway [22]. Although it is known that NIK is necessary for activation of the non-canonical pathway, it was first identified as a kinase which activates the canonical pathway downstream of TNF and IL-1 receptors. Physiological relevance of NIK in the canonical NFκB activation was examined by Zarnegar and coworkers. It was demonstrated that the role of NIK is in the amplification of signals from the canonical pathway. In concert with findings from NIK over-expression studies, NIK activates the canonical pathway when present at high levels due to stabilization [23].

LF was tested for several applications, incl. as newborn babies supplementation [24] or as a feed supplement for newborn piglets [25, 26]. Although there are several producing systems in which LF can be produced as recombinant [27, 28], bovine colostrum still remains as an important source of bioactive constituents incl. LF [29]. Therefore, the aim of our study was to bring additional information about the pathway used by bovine LF free of LPS to stimulate porcine macrophages to the production of proinflammatory cytokines.

## Methods

### Preparation of MDMF from CD14^+^ cells

CD14^+^ porcine monocytes were isolated from whole blood of six 5–6 week–old pigs as described previously [30]. Peripheral blood mononuclear cells (PBMC) were isolated from the collected whole blood by density gradient centrifugation using Histopaque−1077 (Sigma). CD14^+^ monocytes were purified from PBMC by staining with mouse–anti–swine CD14 (clone MIL2, AbD Serotec, Oxford, UK, 10 μl per 10^8^ cells) and goat-anti-mouse IgG MicroBeads followed by an immunomagnetic separation method (QuadroMACS™ cell separator, Miltenyi Biotec, Gladbach, Germany). The cell subset purity was assessed using flow cytometer LSRFortessa^TM^ (BD Biosciences, San Jose, CA) and was more than 95 % in all cases. Purified CD14^+^ monocytes were cultured in DMEM medium supplemented with 10 % fetal bovine serum and 1 % antibiotics (Antibiotic Antimycotic Solution 100×: 10,000 units penicillin, 10 mg streptomycin, and 25 μg amphotericin B per mL; Sigma-Aldrich) for 4 days to differentiate into macrophages (MDMF).

### HEK cells preparation

HEK 293/hTLR4A-MD2-CD14 (HEK4a) cells expressing human TLR4A, MD2 and CD14 molecules and control HEK 293/null (HEK0) cells were obtained from Invivogen (San Diego, USA). Cells were grown in DMEM (Sigma) according to the manufacturer’s recommendation. The cells were seeded onto 24-well plates at the density of 2 × 10^5^ cells per well. A treatment was applied as soon as the cells reached 80 % confluency.

### Purification of lactoferrin

In the study, bovine colostral lactoferrin (Sigma-Aldrich, USA). For part of the study, lactoferrin was purified from LPS. This method was previously described [16]. The interaction between LPS and LF was abrogated by NaCl concentrations higher than 0.4 M [15]. Ten mg of LF (Sigma) was dissolved in 0.5 M NaCl of endotoxin-free water (Sigma), and centrifuged with a 100-kDa Centricon® Plus 70 (Millipore, Centricon, Merck KGaA) to remove endotoxin from the LF. The filtrate, an endotoxin free LF in 0.5 NaCl solution, was desalted by centrifugation with the 10-kDa Centricon® Plus 70. The retentate was recovered, and the concentration was quantified by Pierce BCA Protein Assay Kit 23225 (Thermo Scientific). The success of the purification was estimated by *Limulus* Amebocyte Lysate QCL-1000™ (Lonza). Unpurified and purified LF contained 4.10 EU/ml and 0.05 EU/ml, respectively.

### Iron saturation determination

The level of iron saturation was estimated according to already described method [31]. Briefly, lactoferrin before and after LPS removal was spectroscopicaly measured at 280 and 466 nm with endotoxin-free water (Sigma-Aldrich, USA) as a blank. UV/VIS spectra were obtained using a microplate reader Synergy H1 (Biotek, USA). The acquired A_280_/A_466_ ratios were subsequently used to determine the iron saturation applying the correlation curve established by Majka and coworkers [31]. From accurate measurements by ELISA and ICP-mass spectrometry. The estimation of iron saturation was calculated from an equation:

$$ {\mathrm{A}}_{280}/{\mathrm{A}}_{466} = \mathrm{a}{\left(\mathrm{iron}\ \mathrm{saturation}\right)}^{\mathrm{b}},\ \mathrm{where}\ \mathrm{a} = 933.0\pm 47,\ \mathrm{b} = \hbox{-} 0.817\pm 0.056 $$

Iron saturation was calculated as 64 and 32 % for unpurified and purified LF, respectively.

### Stimulation of HEK and MDMF

In the first experiments, HEK0 and HEK4a cells were treated with 100 μg/ml and 250 μg/ml of lactoferrin to prove the dose-dependent manner. In further experiments, only the dose of 100 μg/ml was used. This dose is sufficient for demonstrable stimulation of cells and is also appropriate because there are significant losses of starting material during purification of lactoferrin. Both types of cells were stimulated with 100 μg/ml unpurified or purified lactoferrin for 6 hours. After stimulation, the cells were harvested. Then, the experiment was stopped by aspiration of the medium and covering cells with RLT buffer with mercaptoethanol (for RT-PCR analysis) or Laemmli buffer (for Western blot analysis). All samples were frozen and stored at −80 °C.

### Quantitative RT-PCR

Total RNA from each well was extracted using RNeasy Mini Kit (Qiagen) according to the manufacturer’s instructions. mRNA was specifically reverse-transcribed using the M-MLV reverse transcriptase system (Invitrogen, Paisley, UK) in the presence of oligo-dT primers. cDNA was diluted 5× and 0.5 μl was used in qPCR. In qPCR analysis, RNA expression was quantified in triplicate reactions in a final volume of 3 μl in 384-well plates using QuantiTect SYBR Green PCR master mix (Qiagen, Hilden, Germany) following the manufacturer’s recommendations, on a LightCycler 480 (Roche Applied Science, http://www.roche.com/). qPCR reactions were prepared with the assistance of Nanodrop II liquid dispenser (Innovadyne Technologies, Rohnert Park, CA). qPCR was performed under the following conditions: denaturation (95 °C for 15 min) and 45 amplification cycles (95 °C for 15 s, 58 °C for 30 s and 72 °C for 30 s). The resulting melting curves were analyzed to test the product specificity. Ten pmol of each primer pair was used per reaction. Primers specific to 4 porcine and 2 human target genes and 2 porcine (HPRT, TBP-1) and 2 human (HPRT, GAPDH) reference genes were used for simultaneous measurements of gene activity (Tables 1 and 2). Among the candidate reference genes, HPRT and GAPDH in the case of MDMF and HEK, respectively, were evaluated as the most constitutively expressed genes in our samples and were selected to adjust mRNA measurements. From the obtained data, relative expression of each target gene was calculated according to the formula [1/(2target gene Ct)]/[1/(2reference gene Ct)].

**Table 1:** Porcine primers used in the study

Gene	Sequence 5′–3′	Reference
CXCL10/IP10	F: CCC ACA TGT TGA GAT CAT TGC	[55]
	R: CAT CCT TAT CAG TAG TGC CG	
HPRT	F: GAG CTA CTG TAA TGA CCA GTC AAC G	[56]
	R: CCA GTC TCA ATT ATA TCT TCA ACA ATC AA	
IL-8	F: TTC TGC AGC TCT TCG TGA GGC	[57]
	R: GGT GGA AAG GTG TGG AAT GC	
NFκB	F: ACG AGC AGA TGG TGA AGG AG	[57]
	R: TCA TGG ATG ATG GCC AAG T	
TNFα	F: CCC CCA GAA GGA AGA GTT TC	[58]
	R: CGG GCT TAT CTG AGG TTT GA

**Table 2:** Human primers used in the study

Gene	Sequence 5′–3′	Reference
GAPDH	F: TCC TAG ATT ATT CTC TGA TTT GGT CGT ATT G	this study
	R: GAA TTT GCC ATG GGT GGA ATC ATA TTG	
IL-8	F: TCC AAA CCT TTC CAC CCC AAA TTT ATC	this study
	R: AGC TCT CTT CCA TCA GAA AGC TTT ACA ATA A	
TNFα	F: CAA TGG CGT GGA GCT GAG AGA TA	this study
	R: CCT TGA AGA GGA CCT GGG ATG AGA T	

### Western blot analysis

For Western blot analysis, MDMF protein lysates were prepared by adding lysis buffer (Cell Signaling Technology) with Laemmli buffer (0.5 M Tris–HCl pH 6.8, glycerol, 10 % SDS, bromophenol blue, beta-mercaptoethanol, deionized water), sonicated and heated to 95 °C for 5 min. Protein lysates were resolved by 10 % SDS-PAGE. After gel electrophoresis, proteins were transferred onto PVDF (polyvinylidene difluoride) membranes. The membranes were blocked in 5 % low-fat dry milk for 1 h at room temperature and followed by incubation with primary antibodies at 4 °C overnight. Primary antibodies used for membrane probing-p-IκB (Ser 32) (Santa Cruz Biotechnology), NIK antibody (Cell Signaling Technology), anti-beta actin antibody (Abcam). Anti-beta actin served as loading control. Next day, the membranes were washed and probed with secondary antibodies for 1 h at room temperature, followed by a washing step. The proteins were visualized on a photographic film, using ECL Plus Western blotting detection reagents (GE Healthcare Life Sciences, UK).

### Statistical analysis

Data were analysed using Statistica 12 (StatSoft). The non-parametric test for paired samples (Wilcoxon’s signed-rank test) was used to detect significant differences among groups. A *p*-value of <0.05 was considered significant.

## Results

### mRNA production of CXCL-10, NFκB inhibitor, IL-8 and TNFα by MDMF after LF treatment

**Fig. 1:**
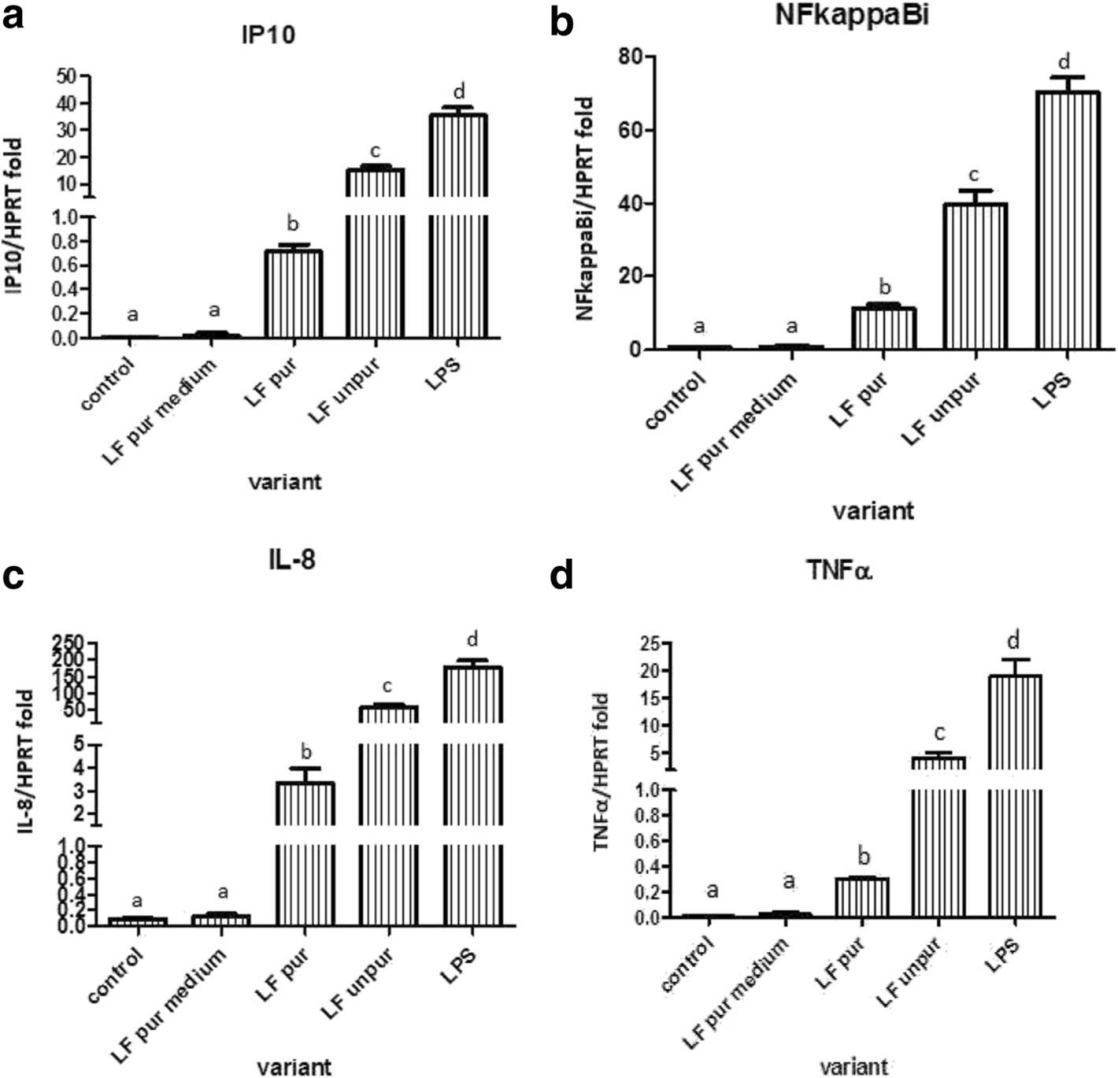
Expression of mRNA for IP10 (**a**), NFκBi (**b**), IL-8 (**c**) and TNFα (**c**) by porcine monocyte-derived macrophages after stimulation with: 1 μg/ml of lipopolysaccharide (LPS); unpurified lactoferrin (LF unpur) and purified lactoferrin (LF pur) containing 4.10 EU/ml and 0.05 EU/ml, respectively. Cells treated with medium containing 1 pg/ml of LPS (i.e. amount comparable with residual LPS-contamination of LF pur) or cultivated in LPS-free medium were used. Data are shown as mean ± SD, *n* = 6. Different letters show statistical significance when compared with control (*p* < 0.05)

Results in Fig. 1a-d suggest that both purified and unpurified LF stimulated MDMF cells. In the case of unpurified LF (i.e., in a complex with LPS), the TLR4-dependent signaling pathway can be expected. In the case of purified LF, the TLR4-dependent pathway can be excluded. This statement is based on results of the experiment in which a medium containing 1 picogram of LPS per milliliter was used (pure LF medium). The concentration was selected because the same residual amount of LPS was detected by the LAL test in the case of the purified form of LF. The results showed that this amount had no impact on the MDMF activation.

### mRNA production of IL-8 and TNFα by HEK0 and HEK4a cells after unpurified LF treatment

**Fig. 2:**
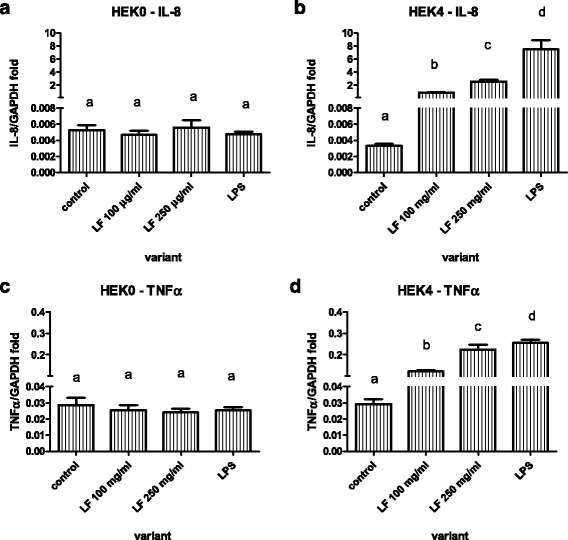
Expression of mRNA for IL8 (**a** and **b**) and TNFα (**c** and **d**) by HEK cell line expressing no TLR (**a** and **c**) or the same cell line expressing TLR4a-MD2-CD14 (**b** and **d**) after stimulation with two concentrations of lactoferrin (LF) or 1 μg/ml of lipopolysaccharide (LPS). Data are shown as mean ± SD of 5 repetitions. Different letters show statistical significance when compared with control (*p* < 0.05)

Activation of the TLR4-dependent pathway was further excluded in experiments using permanent cell lines HEK0 and HEK4a that express either no TLR molecules or TLR4a-MD2-CD14 complex. As expected, stimulation of HEK0 with LPS or with two concentrations of unpurified LF (LF-LPS complex) did not increase expression of mRNA for TNFα and IL-8 (Fig. 2a, c). In the case of HEK4a, this response was induced (Fig. 2b, d).

**Fig. 3:**
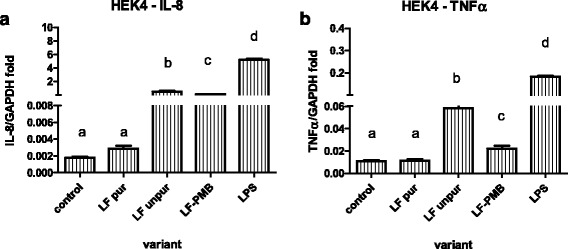
Expression of mRNA for IL8 (**a**) and TNFα (**b**) by HEK cell line expressing TLR4a-MD2-CD14 after stimulation with 1 μg/ml of lipopolysaccharide (LPS); unpurified lactoferrin (LF unpur) and purified lactoferrin (LF pur) containing 4.10 EU/ml and 0.05 EU/ml, respectively. Cells stimulated with unpurified LF in combination with polymyxine B are marked as LF-PMB. Cells cultivated in LPS-free medium were used. Data are shown as mean ± SD of 5 repetitions. Different letters show statistical significance when compared with control (*p* < 0.05)

In contrast to results shown in Fig. 2b and d, expression of mRNA for pro-inflammatory cytokines was not increased when purified LF was used (Fig. 3a, b). The production of IL-8 and TNFα was increased only in the case of unpurified LF and LPS, and it was partially decreased by Polymyxin B (PMB).

### Western blot

**Fig. 4:**
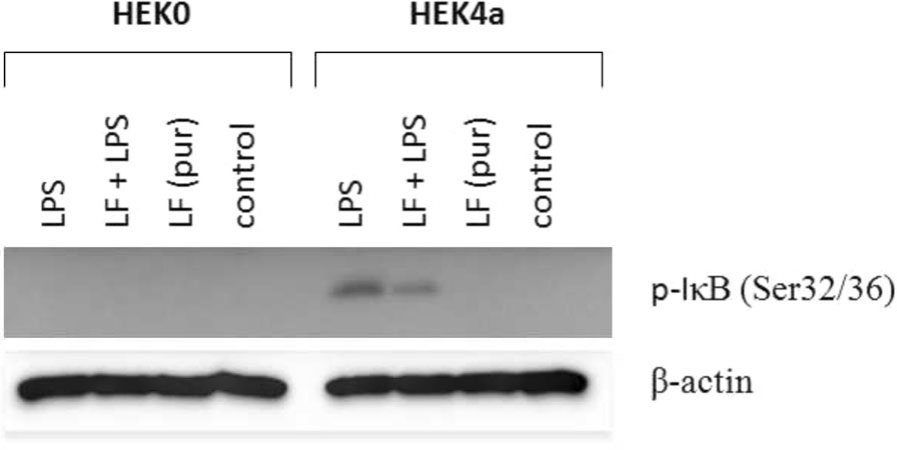
Presence of phosphorylated form of IκB in HEK cell line expressing no TLR (*left*, HEK0) or the same cell line expressing TLR4a-MD2-CD14 (right, HEK4a) after stimulation with (from *left* o *right*) 1 μg/ml of lipopolysaccharide (LPS); 100 μg/ml of unpurified (LF + LPS) or purified lactoferrin (LF pur) containing 4.10 EU/ml or 0.05 EU/ml, respectively. Cells cultivated in LPS-free medium were used as a control. β-actin served as loading control

**Fig. 5:**
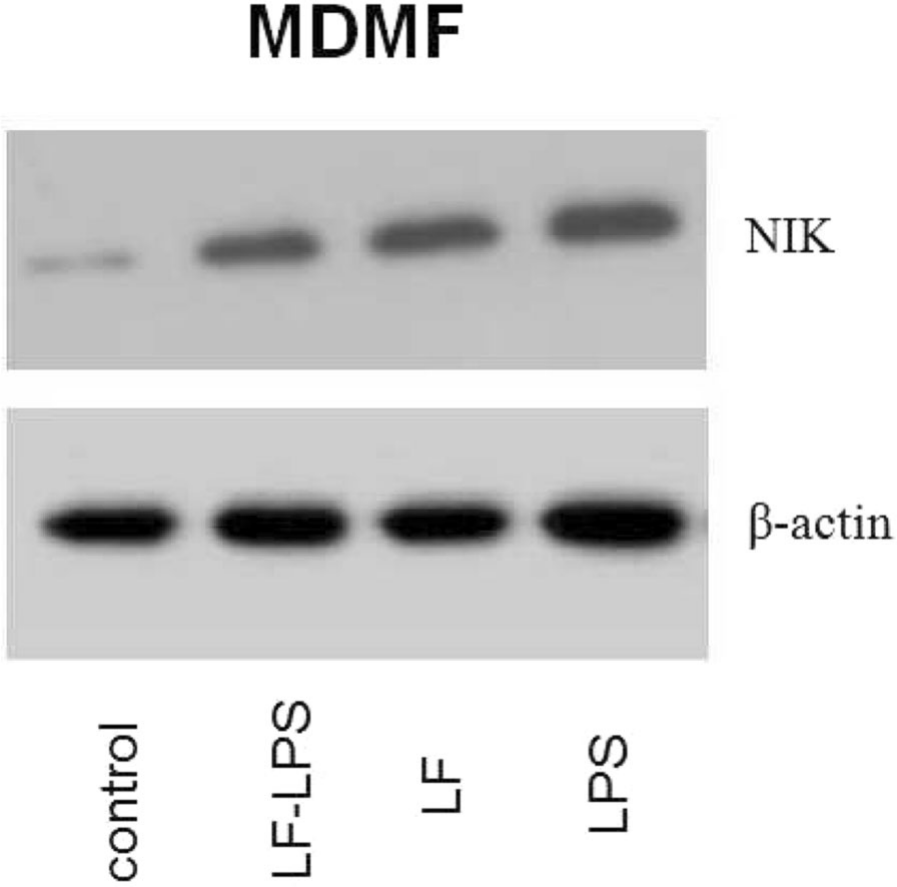
Presence of phosphorylated form of NIK in porcine monocyte-derived macrophages after stimulation with (from *right* to *left*) 1 μg/ml of lipopolysaccharide (LPS); 100 μg/ml of purified (LF) of unpurified lactoferrin (LF-LPS) containing 0.05 EU/ml or 4.10 EU/ml, respectively. Cells cultivated in LPS-free medium were used as a control. β-actin served as loading control

Western blot analysis confirmed that expression of mRNA for pro-inflammatory cytokines in response to LPS alone or unpurified LF (LF-LPS) was conducted via TLR4 and NFκB pathway activation (Fig. 4). Moreover, Western blot analysis also confirmed that MDMF, when stimulated with purified LF, were activated via TLR4-independent pathway. It is clear from the fact that NIK associated with activation of alternative NFκB pathway was present (Fig. 5).

## Discussion

The aim of this study was to extend our knowledge about immunomodulatory properties of LF and particularly about the pathway used by LF free of LPS to stimulate macrophages to the production of proinflammatory cytokines.

As expected, MDMF responded to the stimulation with unpurified LF containing LPS by the production of proinflammatory cytokines. To a lesser extent, these cells were stimulated also by purified LPS-free LF. It suggested that this stimulation is LPS independent so does not use the TLR4 associated pathway. This was confirmed when HEK4a cells were treated with purified LPS-free LF. These cells expressing only TLR4 did not increase the production of IL-8 and TNFa transcripts when compared to control. TLR4-dependent activation was observed only in the case when these cells were stimulated with LF-LPS complex in a dose-dependent manner. This confirmed that LPS-free LF does not activate cells via TLR4 and suggested that it uses an alternative pathway. To confirm that, Western blot analyses were used. Purified LPS-free LF uses an alternative pathway which activates NIK (NF-κB-inducing kinase). NIK forms a complex with phosphorylated IKK1 and IKK2, subsequently leading to the phosphorylation of IκB and translocation of NF-κB to the nucleus [32]. NIK is essential for alternative NFκB pathway, being required for the processing of p100 to p52 [22]. Concerning LPS, it is known that NIK induces phosphorylation of IKK1, that in turn phosphorylates histone H3 [33]. It may be relevant also for LF-LPS (unpurified LF) treatment. Zarnegar and coworkers showed the relevance of NIK in canonical NFκB pathway, when NIK is present at high levels. They demonstrated elevation of canonical NFκB pathway activity on TRAF3−/− mice. In this case NIK was stabilized and present at high levels due to the deficiency of TRAF3 [23]. TRAF3 is an essential negative regulator of alternative NFκB pathway, but in the study it was shown that TRAF3 suppresses canonical NFκB activation and gene expression both in vitro and in vivo. Deregulation of canonical pathway in TRAF3-deficient cells causes NIK accumulation, i.e. TRAF3 inhibition results in coordinated activation of both canonical and alternative pathways [23]. NIK probably lies at a cross road of the canonical and non-canonical pathways of NFκB and it maintains the positive and negative regulatory balance between these two pathways. Maintaining this balance is crucial for the proper physiological responses [32, 34].

LF has no specific receptor on the surface of monocytes/macrophages. It can bind to different low- or high-affinity receptors. The similar route leading to NFкB activation is conducted via RAGE (receptor for advanced glycation endproducts) which is a multiligand receptor expressed on macrophages, neurons, endothelial cells and a variety of tumor cells [35, 36] and TREM-1 (trigerring receptor expressed on myeloid cells 1) a member of the immunoglobulin superfamily which was found on monocytes and neutrophils [37]. Signals which are conducted via RAGE activate two major pathways. First one comprises CDC42/Rac and the second one encompasses MAPKs that finally lead to NFκB activation [38]. TREM-1 is a positive regulator of inflammation [37]. One of the main mechanisms mediating the expression of TREM-1 is activation of NFκB [39]. TREM-1 modulates the activity and availability of key proteins of the TLR4 signaling cascade [40]. TREM-1 influences TLR4 signaling cascade positively by driving TLR4 into the lipid rafts [41]. In other words, signal conducted via TLR4, RAGE and TREM-1 use connected patways and lead to NFκB activation.

LF has the potential to modify the course of an infection [42]. Being immunomodulator, LF is used as adjunct therapeutic which can alter the immune status of the infected host. Bovine LF is widely used as therapeutic agent or food additive in many animal or human models. In the mouse model, it improves host survival modestly concurrent with the decreased serum cytokines [43] or it protects gut integrity in mouse models of LPS endotoxaemia [44]. Its immunomodulatory properties were also confirmed in the influence of low endotoxine bovine milk LF (<0.2 EU/mg) on expression of presentation molecules on bone marrow derived macrophages (BMMs) study. LF modulated the activity of macrophages and their ability to present an antigen and to stimulate T-cells through increased surface expression of antigen presentation and co-stimulatory molecules such as CD80 and CD86. BMMs cultured with bovine LF increased the number of MHC II^+^ cells [45].

In humans, LF is known to be able to prevent the onset of sepsis in infants [46–48]. LF has a high homology among species [49] thus, heterologous molecules of LF are used as a therapeutic agents. Although, the better protective or immunomodulatory effect of homologous recombinant LF could be expected, it was not proven, concretely in the mouse model of methicillin-resistant *Staphylococcus aureus* challenge. Recombinant mouse LF (rmLF) and recombinant human LF (rhLF) had a high degree of overlap to modulate inflammatory responses. Both recombinant molecules caused a decrease in inflammatory cytokine production in monocytes and an increase of cytokine production in granulocytes during the infection. But the two heterologous recombinant molecules showed some differences in their activities concerning cellular population that were used in the studies-mouse granulocytes demonstrated a different cytokine pattern upon the infection than human granulocytes. LFs were produced under endotoxin free serum conditions, and further affinity purified to remove traces of endotoxin [42]. The data show that endotoxin-free LF has not only immunomodulatory effects but also the therapeutic effects which have different essence than just LPS binding.

A similar study in which low endotoxin human milk LF (<10EU/mg) was used supports a therapeutic significance of LF in vivo. LF treatment reduced TNF-α and IL-6 in serum of LPS-challenged mice during LPS-induced endotoxaemia, when it was delivered to naive mice 1 h prior to administration of LPS. The same molecule showed a prophylactic effect of treatment with LF 18 h prior to LPS-induced endotoxaemia when the production of TNF-α but not IL-6 was affected [50].

## Conclusion

Our results suggest that LF’s ability is not only to bind LPS, but LF itself may be a stimulant of pro-inflammatory pathways. LF’s ability to bind LPS from an environment is known very well [16, 51]. This binding is its natural property which is used for example against bacterial infections. Due to this fact, LF often occurs in an unpurified form in a complex with LPS [18]. This kind of “contamination” resulting from one of the natural properties of LF could lead to some overvalued results. It should be considered when studying the production of cytokines or chemokines after LF treatment. Similar situations were found concerning other therapeutic proteins. When testing ovalbumin, it was found that a purified form of this protein did not activate endothelial cells in vitro. If an unpurified commercial ovalbumin containing LPS was used, endothelial cells were activated in early stages of inflammation [52]. False pro-inflammatory effects of commercial alpha-lactalbumin were previously described in RAW 264.7 macrophages due to endotoxin contamination [53]. And, finally, other authors mentioned contamination of commercial LF [18, 54]. This fact should be borne in mind when we perform experiments in which we study pro-inflammatory parameters.

## Abbreviations

HEK: Human embryonic kidney
IL: Interleukin
IκB: Inhibitor of NF-kappa-B
LF: Lactoferrin
LPS: Lipopolysaccharide
MDMF: Monocyte-derived macrophages
mRNA: Messenger ribonucleic acid
NFκB: NF-kappa-B
NIK: NF-kappa-B-inducing kinase
RT-PCR: Real-time polymerase chain reaction
TLR: Toll-like receptor
TNF: Tumor necrosis factor
